# Deep learning method for prediction of patient-specific dose distribution in breast cancer

**DOI:** 10.1186/s13014-021-01864-9

**Published:** 2021-08-17

**Authors:** Sang Hee Ahn, EunSook Kim, Chankyu Kim, Wonjoong Cheon, Myeongsoo Kim, Se Byeong Lee, Young Kyung Lim, Haksoo Kim, Dongho Shin, Dae Yong Kim, Jong Hwi Jeong

**Affiliations:** 1 Department of Radiation Oncology, Proton Therapy Center, National Cancer Center, 323, Ilsan-ro, Ilsandong-guGyeonggi-do, Goyang-si, 10408 South Korea; 2 Department of Radiation Oncology, Yonsei Cancer Center, Yonsei University College of Medicine, 50-1 Yonsei-ro, Seodaemun-gu, Seoul, 03722 South Korea

**Keywords:** Deep learning, Dose prediction, Volumetric modulated arc therapy (VMAT), Knowledge-based planning (KBP), RapidPlan™

## Abstract

**Background:**

Patient-specific dose prediction improves the efficiency and quality of radiation treatment planning and reduces the time required to find the optimal plan. In this study, a patient-specific dose prediction model was developed for a left-sided breast clinical case using deep learning, and its performance was compared with that of conventional knowledge-based planning using RapidPlan™.

**Methods:**

Patient-specific dose prediction was performed using a contour image of the planning target volume (PTV) and organs at risk (OARs) with a U-net-based modified dose prediction neural network. A database of 50 volumetric modulated arc therapy (VMAT) plans for left-sided breast cancer patients was utilized to produce training and validation datasets. The dose prediction deep neural network (DpNet) feature weights of the previously learned convolution layers were applied to the test on a cohort of 10 test sets. With the same patient data set, dose prediction was performed for the 10 test sets after training in RapidPlan. The 3D dose distribution, absolute dose difference error, dose-volume histogram, 2D gamma index, and iso-dose dice similarity coefficient were used for quantitative evaluation of the dose prediction.

**Results:**

The mean absolute error (MAE) and one standard deviation (SD) between the clinical and deep learning dose prediction models were 0.02 ± 0.04%, 0.01 ± 0.83%, 0.16 ± 0.82%, 0.52 ± 0.97, − 0.88 ± 1.83%, − 1.16 ± 2.58%, and − 0.97 ± 1.73% for D_95%_, D_mean_ in the PTV, and the OARs of the body, left breast, heart, left lung, and right lung, respectively, and those measured between the clinical and RapidPlan dose prediction models were 0.02 ± 0.14%, 0.87 ± 0.63%, − 0.29 ± 0.98%, 1.30 ± 0.86%, − 0.32 ± 1.10%, 0.12 ± 2.13%, and − 1.74 ± 1.79, respectively.

**Conclusions:**

In this study, a deep learning method for dose prediction was developed and was demonstrated to accurately predict patient-specific doses for left-sided breast cancer. Using the deep learning framework, the efficiency and accuracy of the dose prediction were compared to those of RapidPlan. The doses predicted by deep learning were superior to the results of the RapidPlan-generated VMAT plan.

## Background

As radiation therapy treatment technology is advancing, the treatment outcomes of cancer patients are gradually improving. Recently, advanced treatment modalities, such as intensity modulation radiation therapy (IMRT) and volumetric arc therapy (VMAT), have been applied to deliver higher doses to tumor areas, while reducing the therapeutic doses to normal organs compared to conventional 3D conformal radiation therapy. However, the ability of these advanced treatment methods to produce an optimal plan varies according to the experience of the planner, and a planning time-consuming task must be repeated until the treatment goal is reached. Therefore, studies [1–6] have been conducted to improve treatment planning efficiency and quality, while reducing planning time and effort by using knowledge-based techniques for dose prediction in radiotherapy.

Currently, commercial software is available in the form of RapidPlan (version 13.6, Varian Oncology Systems, Palo Alto, CA, USA) [7–9]. A knowledge-based planning (KBP) model is generated using a previous, clinically approved treatment plan data-based regression analysis as the dose-volume histogram (DVH) estimation algorithm of RapidPlan. For a new patient, the most similar treatment plan is provided within the estimated DVH model.

However, dose prediction using the KBP model has two limitations. First, it is difficult to include all characteristics of the inherent organ structure depending on the patient. Second, the DVH does not reflect the spatial dose distribution. It is possible to derive an unacceptable plan according to the dose distribution around an important organ at risk (OAR). If patient-specific dose prediction were possible while compensating for the limitations of the KBP model, the workload in clinical practice would be reduced.

Deep learning methods have proven to be effective in various fields, such as automatic segmentation [10–12], image registration [13–15], respiratory motion prediction [16–18], and toxicity prediction [19–21]. Studies on dose prediction using deep learning have also been reported.

Deep learning methods based on dose prediction of IMRT plans have been utilized for head and neck [22, 23], rectal [24], prostate [25, 26], and lung [27] cancer cases. In addition, dose prediction using VMAT plans has been performed for head and neck [28], rectal [29], and prostate [30] cancer. There have also been other treatment techniques such as 3D dose prediction for head and neck cancer treatment using helical tomotherapy [31].

In those studies, evaluation of the test cases was conducted after learning a deep neural network using 2D or 3D computed tomography (CT) images and patient anatomical information. However, when evaluating the accuracies of the deep learning models, comparisons were not made under the conditions used in the commercial models; rather, only the DVH, mean dose, maximum dose, and gamma index [32] were employed for quantitative evaluation of the clinically accepted plan.

In this study, the doses predicted by developed deep learning model using only the anatomy information of left-sided breast cancer patients treated with VMAT were compared with the results predicted using RapidPlan under the same clinically acceptable conditions.

## Methods

### Patients and treatment planning

Fifty-five patients with left-sided breast cancer diagnosed at the National Cancer Center in South Korea in 2018 and 2019 were included in this study. The characteristics of the patients are listed in Table 1. All patients were staged according to the American Joint Committee on Cancer (AJCC) staging system [33].

**Table 1:** Patient characteristics in this study

Patients	Female	Average age	Average left breast volume [cm^3^]	Stage				
				I	II	III	IV	N/X
Training set	45	51	438.15	35	6	-	-	4
Testing set	10	56	491.51	7	3	-	-	-

Manual planning and treatment were performed for all patients with two coplanar VMAT arcs with a prescription of 4320 cGy in 16 fractions, a photon energy of 6 MV beam, gantry angles of 165°–290°, and collimator angles of 30° and 330°. The entire breast was included, while there was no nodal involvement.

All treatments were planned using the Varian Eclipse treatment planning system (version 13.6, Varian Oncology Systems, Palo Alto, CA, USA) with the analytical anisotropic algorithm (AAA). Table 2 shows the planning goals for the PTV, heart, left lung, right lung, and right breast.

**Table 2:** Left-sided breast cancer clinical treatment planning goals

Target and normal organ structure	Planning goals
Planning target volume (PTV)	Dose 95 [%] = 100
	Dose [max] ≤ 49.68 Gy
Heart	Dose [mean] ≤ 4 Gy
	Volume 5 [%] ≤ 20 Gy
	Volume 30 [%] ≤ 10 Gy
Lung (left, right)	Volume 15 [%] ≤ 20 Gy
	Volume 35 [%] ≤ 10 Gy
	Volume 50 [%] ≤ 5 Gy
Right breast	Dose [max] ≤ 5 Gy

*Volume [% of total volume]

The clinical treatment plan was optimized to ensure the following OAR constraints: no more than 5% of the heart received > 20 Gy (Volume 5 [%] ≤ 20 Gy); no more than 30% of the heart received > 10 Gy (Volume 30 [%] ≤ 10 Gy); no more than 4 Gy of the heart mean dose received; no more than 15% of the lungs received > 20 Gy (Volume 15 [%] ≤ 20 Gy); no more than 35% of the lungs received > 10 Gy (Volume 35 [%] ≤ 10 Gy); no more than 50% of the lungs received > 5 Gy (Volume 50 [%] ≤ 5 Gy); no more than 5 Gy of the right breast max dose received. All the clinical confirmation plans were normalized such that 95% of the PTV received 100% of the prescription dose. For each plan, the contours of the PTV and OARs were determined by experienced physicians, and the dose distribution was confirmed by experienced physicians and physicists. The same datasets were used in the RapidPlan method for comparison.

All CT images were acquired using General Electric Light Speed Radiotherapy System 4 (GE Medical Systems, Milwaukee, WI). CT images with the following dimensions were utilized for each axial slice: image matrix = 512 × 512, slice numbers = 76–106, pixel spacing = 0.98 mm, and slice thickness = 3.75 mm. The study protocol conformed to the ethical guidelines of the Declaration of Helsinki as revised in 1983 and was approved by the Institutional Review Board (IRB) of the National Cancer Center without an IRB number. All patient data were fully anonymized, and all methods were performed in accordance with the relevant guidelines and regulations outlined by our institution.

### Deep learning model for dose prediction

**Fig. 1:**
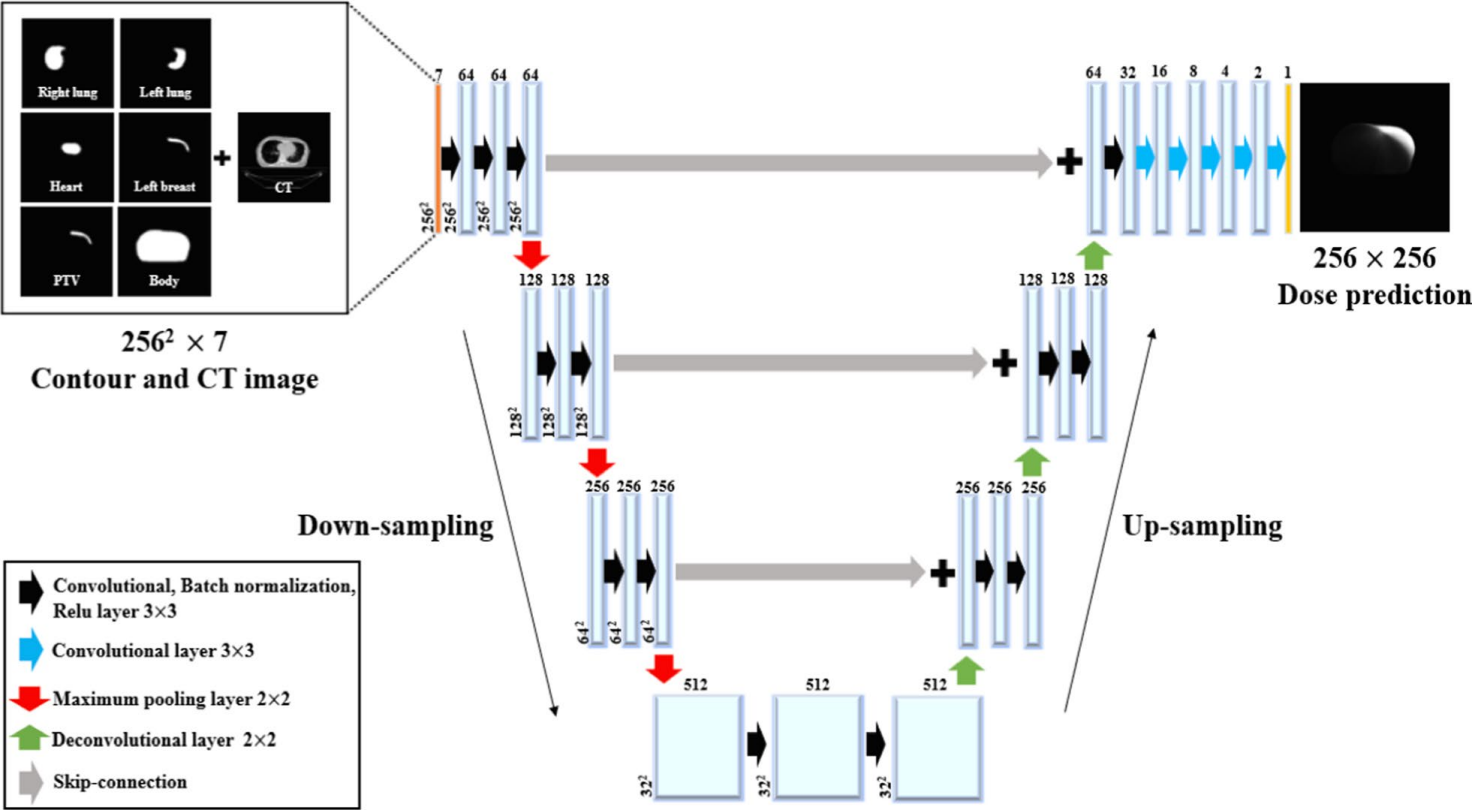
Schematic diagram of the U-net based dose prediction deep neural network (DpNet) architecture used for volumetric arc radiation therapy (VMAT) dose distribution prediction

The network used was based on the open-source library Keras (version 2.2.4) [34] and the reference implementation of U-Net [35]. Figure 1 shows the dose prediction deep neural network (DpNet) for dose prediction, which consists of a down-sampling (encoding) path and an up-sampling (decoding) path. For the encoding path, we used two 3 × 3 convolution layers, which had 64, 128, 256, and 512 filters. Each of these layers was followed by a rectified linear unit (ReLu) [36] and a maximum pooling layer. On the decoding path, we used a 2 × 2 transposed convolution and two 3 × 3 convolution layers followed by a ReLu activation function. Concatenation was performed with the corresponding feature map from the skip connection path and two convolution layers with 3 × 3 filters. To avoid overfitting during training, batch normalization [37] and dropout (the dropout rate was set to 0.2) [38] were added to the layers. In the final layer, we used a 1 × 1 convolution network with a sigmoid activation function. The mean squared error (MSE) loss function utilized in DpNet calculates the difference between the actual and predicted doses according to Eq. (1):

1 $$MSE = \frac{1}{n}\mathop \sum \limits_{i = 1}^{n} (D_{p}^{i} - D_{c}^{i} )^{2} ,$$

where n is the total number of training samples and p and c are the predicted and clinical doses, respectively. We used Adam [39] as an optimizer with a learning rate of 1.0E-04 and mini-batch size of 15 images. The experiments were conducted on a computer workstation with an Intel i7 central processing unit with a 24 GB main memory and a computer unified device architecture library on a graphics processing unit (NVIDIA Titan-Xp with 12 GB of memory). Network training of the DpNet took approximately 48 h to run 5000 epochs on the training and validation datasets.

### Dose prediction data preprocessing

The input of the dose prediction model was utilized for training and validation of the DpNet, including CT images as well as PTV, heart, left lung, right lung, and right breast contour images. Clinical plan datasets were obtained using Eclipse planning software (version 13.6, Varian Oncology Systems, Palo Alto, CA, USA). All CT images were converted into grayscale images, and the contouring points were converted into segmented contour images in binary format, as depicted in Fig. 1 [12]. All training images were resized from the conventional size of 512 × 512 pixels to 256 × 256 pixels owing to graph card memory resource limitations and to reduce the DpNet training time.

### Deep-learning-based dose prediction process

**Fig. 2:**
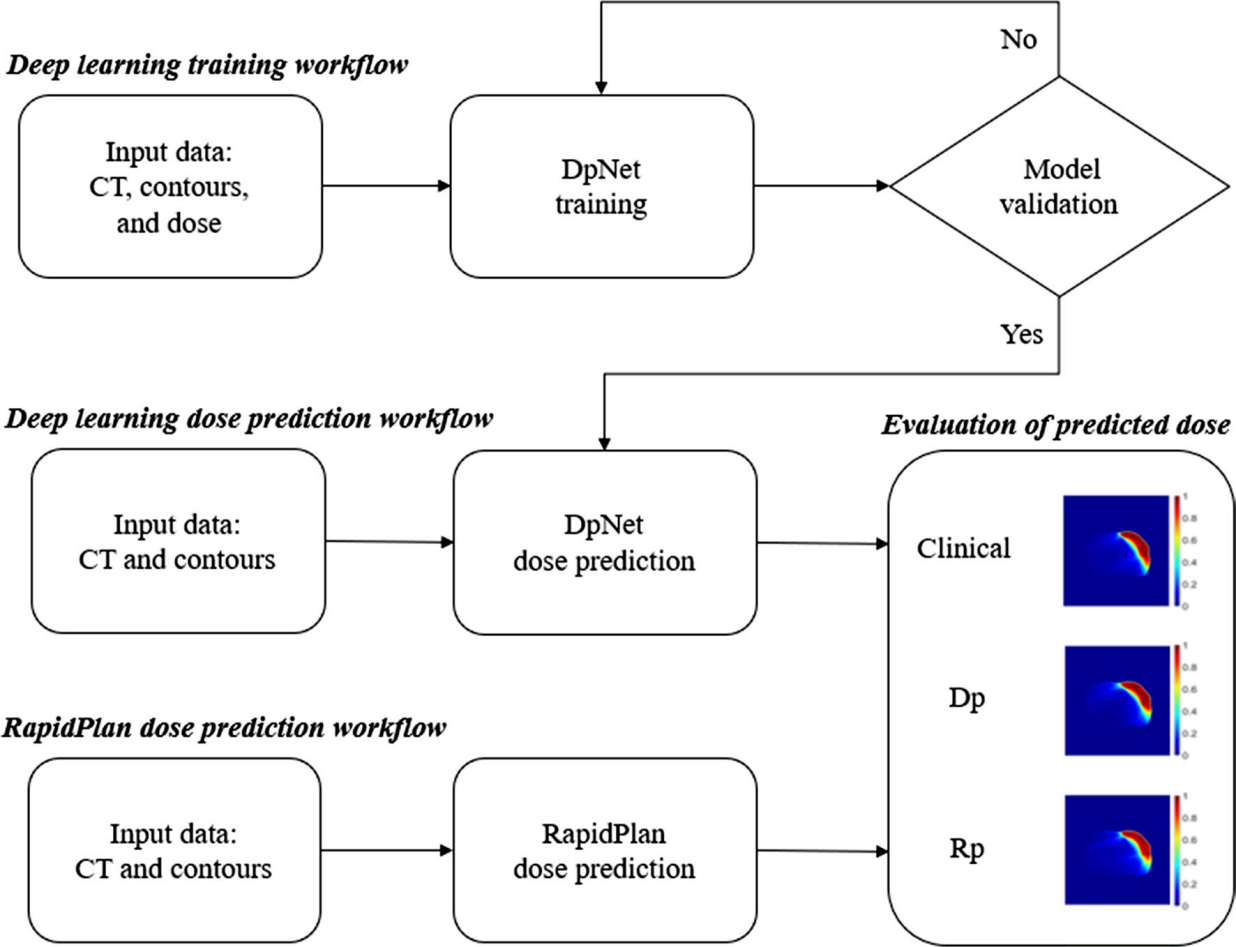
Flowchart showing the deep learning dose prediction (Dp) and Rapid plan dose prediction (Rp) process.

The deep learning dose prediction (Dp) model is a DpNet training of contours, a CT image as input data, and the training and validation sets consisted of 35 and 10 patient datasets, respectively. The 10 test sets used for model validation were employed as independent, separate dataset images for DpNet, as shown in Fig. 2.

Five-fold cross-validation [40] was performed to improve the model accuracy because the training dataset was insufficient. The five-fold average loss ± standard deviation was 0.09 ± 0.01 (training loss) and 0.73 ± 0.07 (validation loss). The third fold performed the best, with the lowest validation loss of 0.65. This model was used to evaluate the dose predictions for the test set of patients. To compare the results with the clinical dose distribution, the predicted dose of the test set was normalized to 100% of the prescription dose in 95% of the PTV. For the dose prediction of the Dp model, the test process is carried out with the patient CT as an input, and dose prediction is performed not only on the PTV contour but also on the area that does not include the PTV contour as the entire area inside the body contour.

### RapidPlan model for dose prediction

The RapidPlan dose prediction (Rp) model configuration consisted of two parts.

First, the geometric and dosimetric information of the patient was extracted from a group of selected available approved treatment plans (training and validation datasets), and an automated DVH estimation model was created based on the extracted features.

Second, the lower boundary of the DVH estimation model parameter of the new patient (test dataset) was generated by predicting the DVH using the trained, optimized model.

To compare and evaluate the performance of the Dp, the dose distribution was generated using RapidPlan with the same training, validation, and test datasets. AAA (version 13.6) was used as the dose calculation algorithm, and normal tissue objects were not used in the same way as in the clinical plan. All normalized dose distributions were the same as those in the clinical protocol of our institution, as mentioned above.

For PTV, the manual optimization objectives were applied in the same way as in the clinical plan; separately, the dose volume constraints were applied as OAR optimization objectives to the line objectives generated from the predicted DVH. The Rp plan is a result of the optimization process; thus, the similarity between the original plan and the Rp plan depends on the similarity of the objective setting in the original plan and in the Rp plan creation.

### Quantitative dose prediction evaluation

To evaluate quantitatively the accuracy of Dp and Rp, the 3D dose distribution, the maximum and mean dose absolute differences between the clinical and predicted doses in the OARs and PTV, the DVH, 2D gamma analysis, and the isodose volume dice similarity coefficient (iDSC) [31] were used.

First, the clinical and predicted dose volumes of the Dp and Rp models were compared with the 3D dose distribution.

Second, in the indirect evaluation method, the absolute dose errors of the clinical and predicted doses were calculated using the following equation:

2 $$PTV,\;OARs\;percentage\;of\;absolute\;error = \left| {\frac{{Clinical\;dose - Predicted\;dose\left( {Dp,\;Rp} \right)}}{Clinical\;dose}} \right| \times 100\% .$$

Third, the clinical and predicted doses obtained using the DVH, the most commonly used treatment plan evaluation tool, were compared.

Fourth, the gamma analysis metric, which is utilized in evaluating complex modulated radiotherapy, was calculated by simultaneously considering the dose difference and distance to agreement. The clinical dose was compared with the 2D gamma index as a reference, and the 2D dose distribution corresponding to the transverse plane was calculated. The gamma index passing criteria were 3%/3 mm and 2%/2 mm, and the calculation for the whole body was performed without a dose threshold. In addition, areas outside the body were not included in the gamma index calculation.

Finally, the dice similarity coefficient of the isodose volume was evaluated in the 3D dose distribution. The iDSC method involves calculating the overlapping results of two different volumes according to the following equation:

3 $${\text{iDSC}} = \frac{{2\left| {A \cap B} \right|}}{\left| A \right| + \left| B \right|},$$

where A is the clinical isodose volume and B is the predicted isodose volume (Dp and Rp). iDSC takes values between zero and one. When iDSC approaches zero, the clinical and prediction results differ significantly. However, as iDSC approaches one, the two volumes exhibit increased similarities.

We used the Wilcoxon test to determine the statistical significance of the differences between the clinical Dp and Rp results.

## Results

**Fig. 3:**
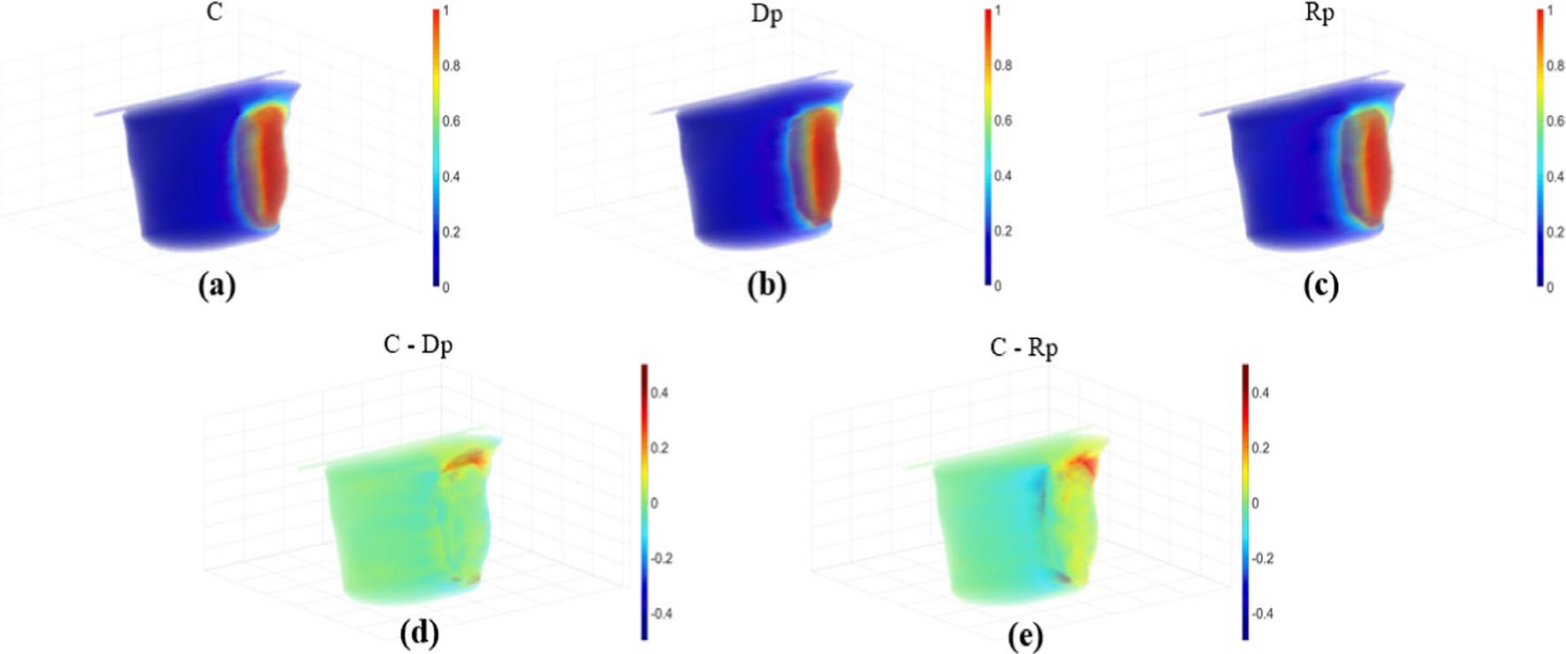
An example of a left-sided breast cancer 3D-dose distribution illustrating the prediction accuracy, No.4 patient case. **a**–**c** Illustrate clinical (**c**), deep learning dose prediction (Dp), and Rapid plan dose prediction (Rp) relative dose distributions for each voxel, **d** relative dose difference between clinical and Dp, **e** relative dose difference between clinical and Rp

**Fig. 4:**
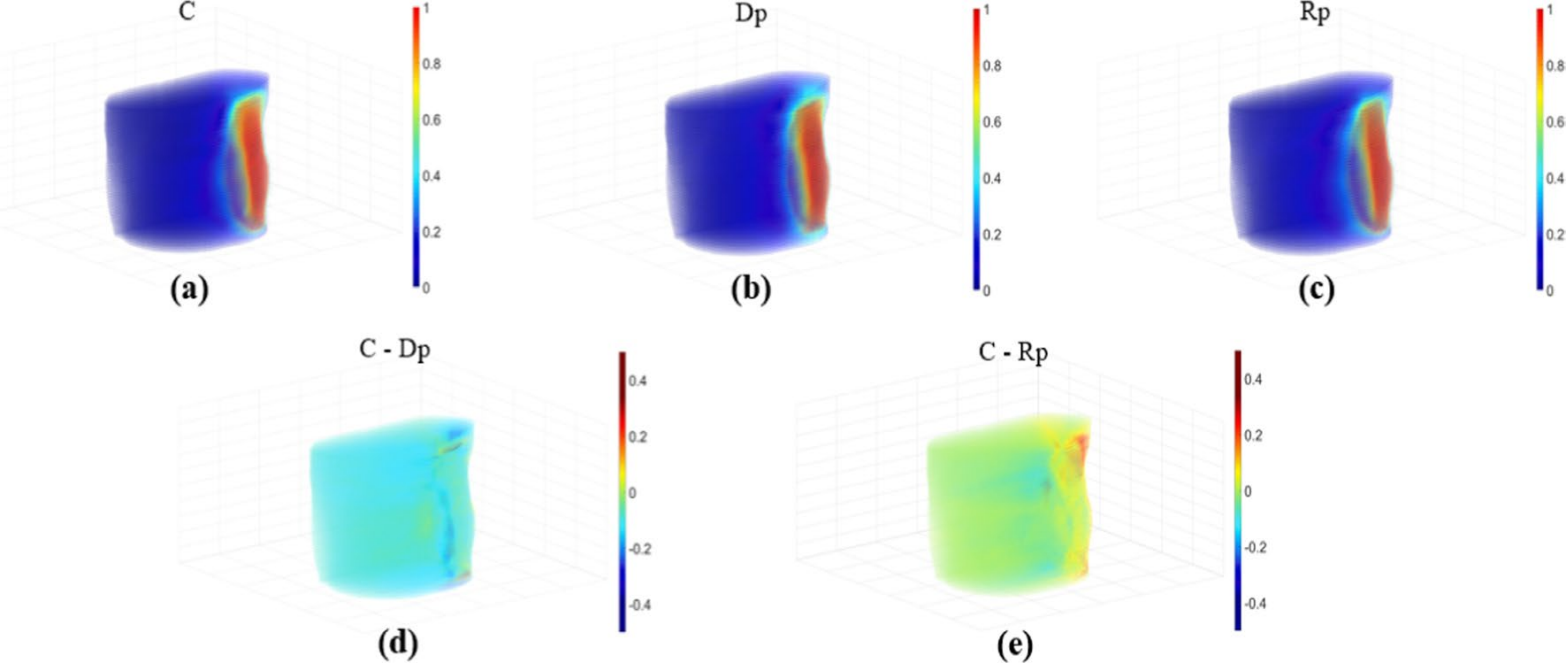
An example of a left-sided breast cancer 3D-dose distribution illustrating the prediction accuracy, No.8 patient case. **a**–**c** illustrate clinical (**c**), deep learning dose prediction (Dp), and Rapid plan dose prediction (Rp) relative dose distributions for each voxel, **d** relative dose difference between clinical and Dp, **e** relative dose difference between clinical and Rp

**Fig. 5:**
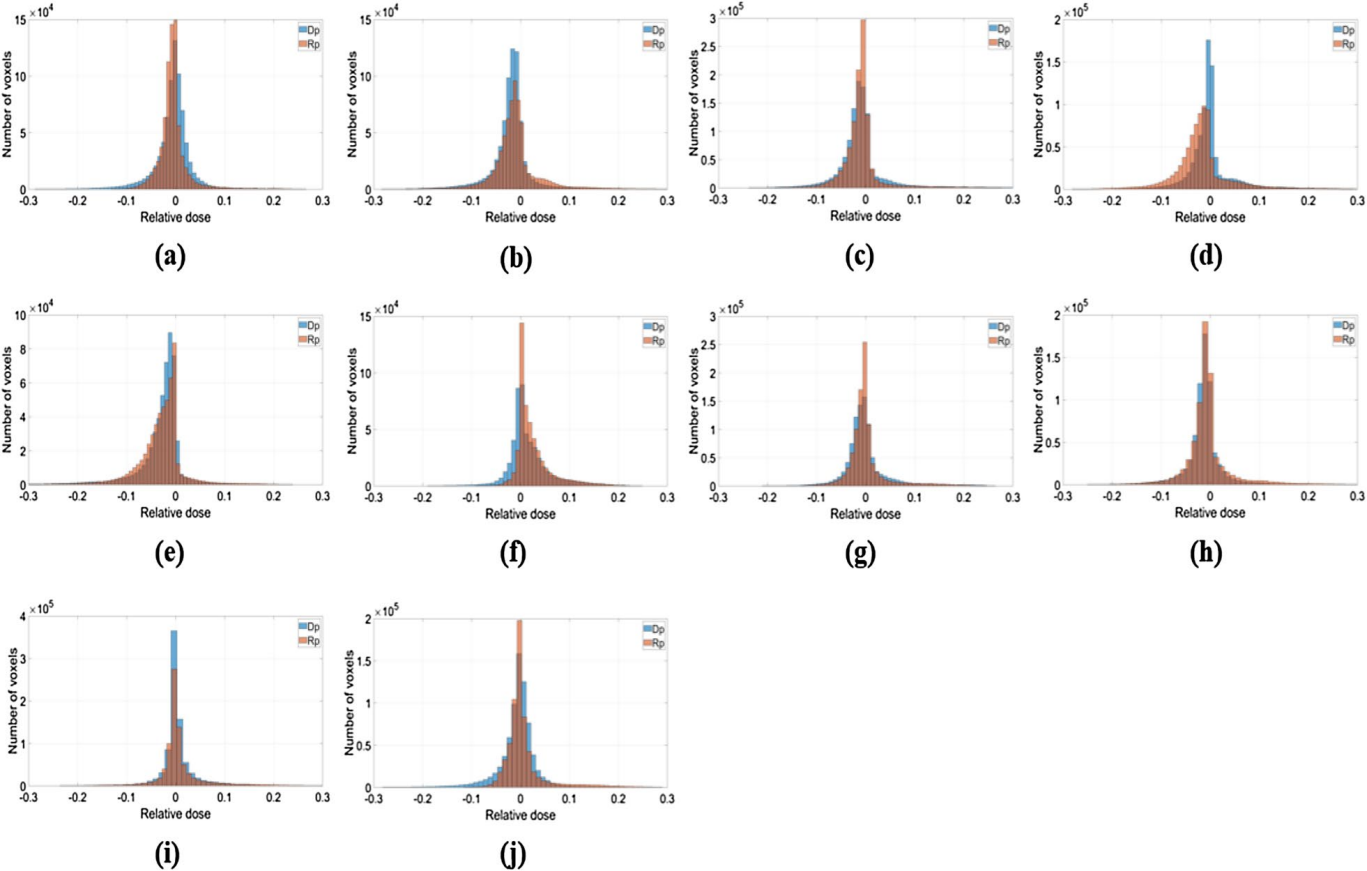
The relative 3D-dose distribution difference compared clinically with the Dp and Rp is shown by histogram for the ten-test data set. **a**–**j** are test set cases of No. 1–10. Zero value of relative dose was not included in the histogram plot

For quantitative evaluation, Figs. 3, 4 and 5 depict the relative dose differences for the test set between the clinical dose and the Dp and Rp 3D dose distributions and the differences from the clinical dose histogram. The histogram distributions were compared; in cases 2, 4, and 5 of the test set, the Dp dose showed less difference from the clinical dose than the Rp dose. In cases 1, 6, and 10 of the test set, the Rp dose was more consistent with the clinical dose than the Dp dose, and there was no significant difference in cases 7–9, as demonstrated in Fig. 5.

Tables 2 and 3 present the absolute maximum and mean dose errors and SDs of the PTV and organ structures of the test set. For the absolute maximum dose error, the average differences between the errors of the Dp and Rp are 1.28%, 0.90%, − 3.74%, − 4.24%, and − 3.07% for the body, left breast, heart, left lung, and right lung, respectively, as shown in Table 3. For the absolute mean dose error, the average differences between the Dp and Rp are –0.13%, –0.78%, 0.56%, 1.04%, and –0.77% for the body, left breast, heart, left lung, and right lung, respectively, as summarized in Table 3. In the PTV case, the differences in in D_95%_, D_50%_, D_2%_, and D_mean_ between the Dp and Rp models are less than 1%, although the difference in D_max_ is larger, as observed in Table 4.

**Table 3:** Comparative mean absolute error average (± SD) dosimetric results for OARs in 10 tested patients

Organ structure	D_mean_				D_max_			
	C-Dp (a) (%)	C-Rp (b) (%)	*p* value	$\left| {\left| a \right| - \left| b \right|} \right|$(%)	C-Dp (a) (%)	C-Rp (b) (%)	*p* value	$\left| {\left| a \right| - \left| b \right|} \right|$(%)
Body	0.16 ± 0.82	− 0.29 ± 0.98	0.01	− 0.13	2.21 ± 2.14	0.93 ± 1.77	0.01	1.28
Left breast	0.52 ± 0.97	1.30 ± 0.86	0.01	− 0.78	2.08 ± 2.13	1.18 ± 1.92	0.13	0.9
Heart	− 0.88 ± 1.83	− 0.32 ± 1.10	0.20	0.56	− 1.79 ± 8.43	5.53 ± 4.97	0.01	− 3.74
Left lung	− 1.16 ± 2.58	0.12 ± 2.13	0.05	1.04	0.67 ± 3.71	4.91 ± 2.80	0.01	− 4.24
Right lung	− 0.97 ± 1.73	− 1.74 ± 1.79	0.25	− 0.77	− 6.73 ± 9.13	− 9.8 ± 10.03	0.16	− 3.07

**Table 4:** Comparative mean absolute error average (± SD) dosimetric results for PTV in 10 tested patients

Dosimetric index	PTV			
	C-Dp (a) (%)	C-Rp (b) (%)	*p* value	||*a*|–|*b*|| (%)
D_95%_	0.02 ± 0.04	0.02 ± 0.04	–	0
D_50%_	0.86 ± 0.53	0.90 ± 0.72	0.75	− 0.04
D_2%_	1.01 ± 0.67	0.91 ± 0.53	0.50	0.1
D_max_	1.51 ± 2.68	0.58 ± 1.82	0.08	0.93
D_mean_	0.01 ± 0.83	0.87 ± 0.63	0.01	− 0.86

D_n%_ is means the dose received by n % of the PTV

**Fig. 6:**
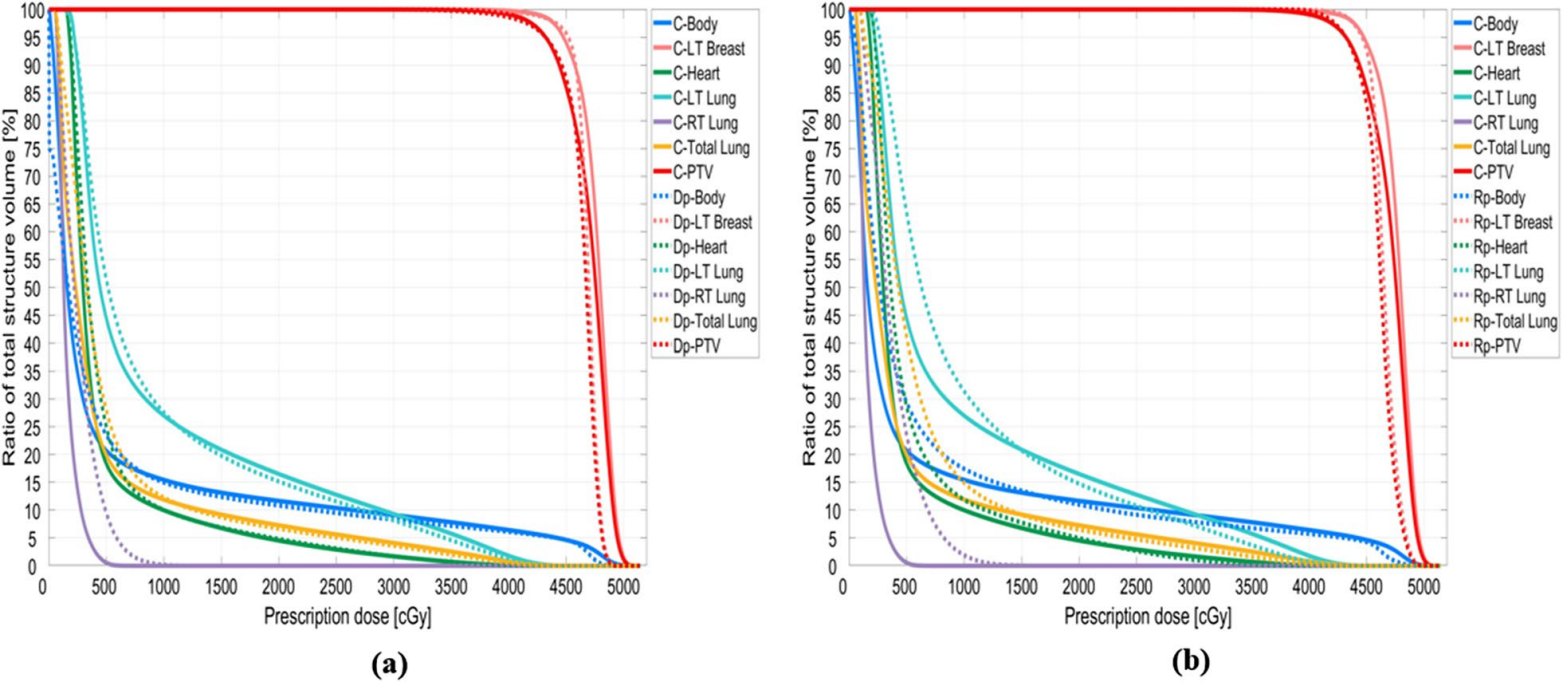
No.4 patient case, comparison of deep-learning, and rapid-plan dose-volume histogram, solid lines represent clinical DVH, and dashed lines represent predicted DVH, **a** compare DVH results of clinical and Dp, **b** compare DVH results of clinical and Rp

**Fig. 7:**
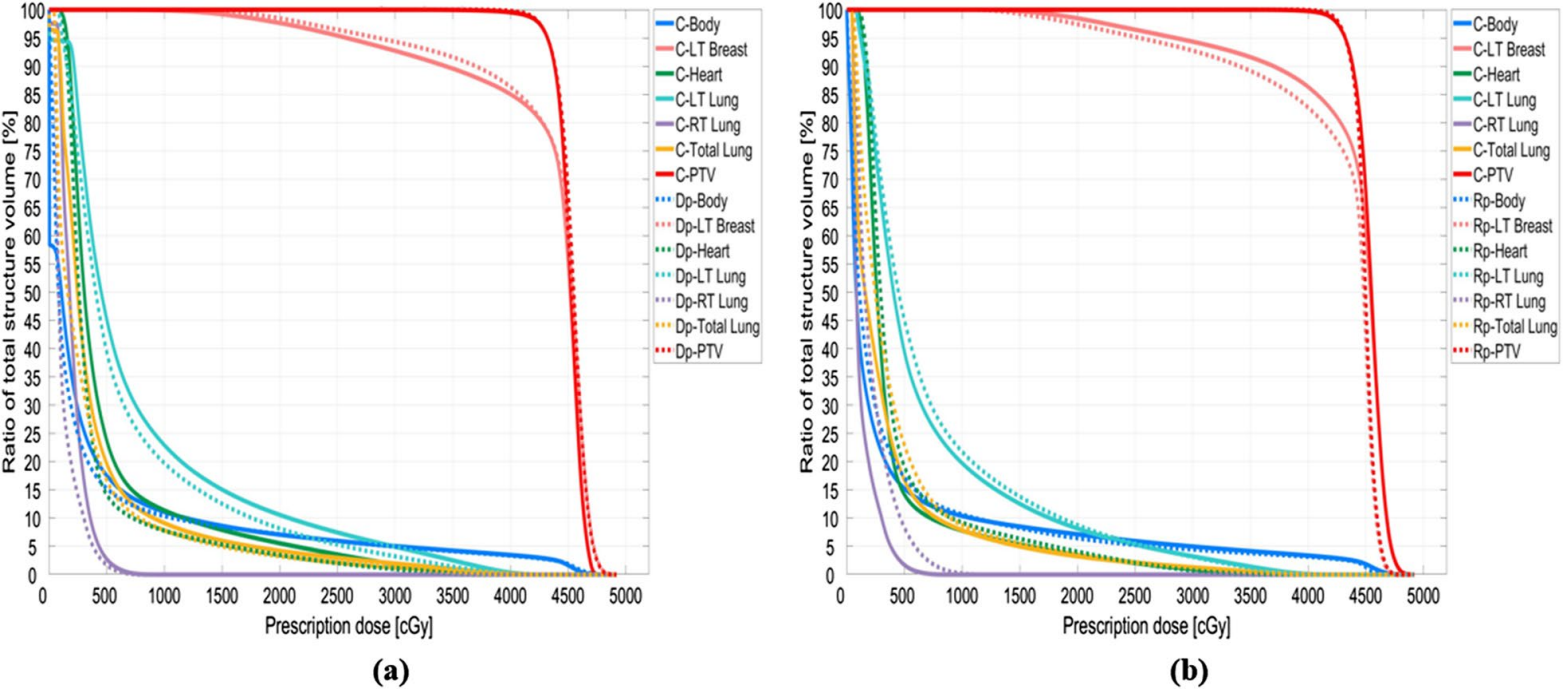
No.8 patient case, comparison of deep-learning, and rapid-plan dose-volume histogram, solid lines represent clinical DVH, and dashed lines represent predicted DVH, **a** compare DVH results of clinical and Dp, **b** compare DVH results of clinical and Rp

**Fig. 8:**
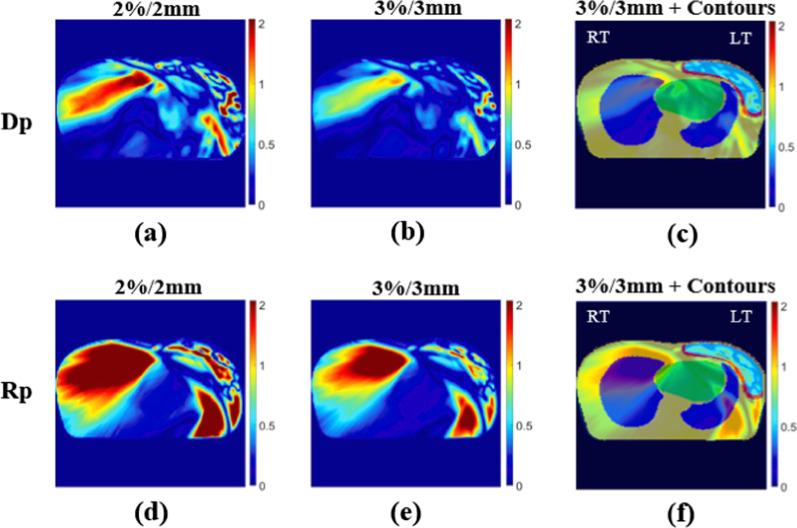
2D gamma analysis results of No.4 patient case at isocenter position, **a** dose difference and distance to agreement passing criteria of 2 %/2 mm Dp, **b** criteria of 3 %/3 mm Dp, **d** criteria of 2 %/2 mm Rp, **e** criteria of 3 %/3 mm Rp, **c**, **f** 3%/3mm gamma analysis results and contours overlapping images (the meaning of contour color, yellow: body, green: heart, blue: lungs, red: left breast, cyan: PTV)

**Fig. 9:**
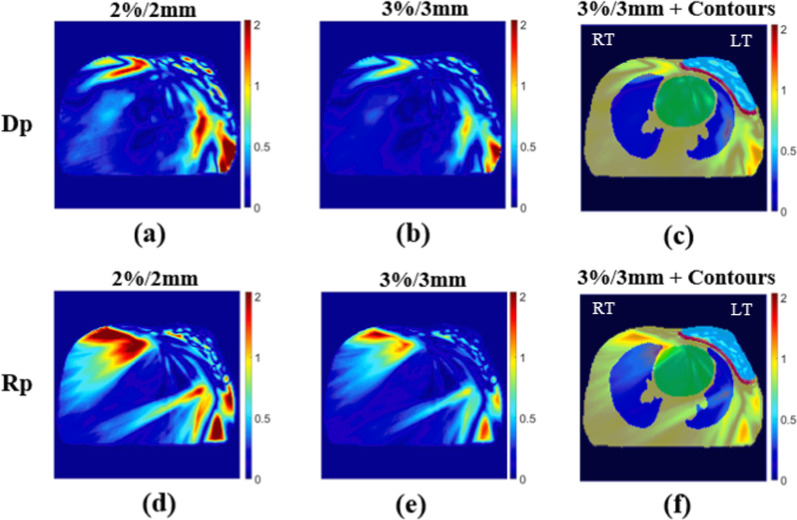
2D gamma analysis results of No.8 patient case at isocenter position, **a** dose difference and distance to agreement passing criteria of 2 %/2 mm Dp, **b** criteria of 3 %/3 mm Dp, **d** criteria of 2 %/2 mm Rp, **e** criteria of 3 %/3 mm Rp, **c** and **f** 3%/3mm gamma analysis results and contours overlapping images (the meaning of contour color, yellow: body, green: heart, blue: lungs, red: left breast, cyan: PTV)

The DVHs of two patients (Nos. 4 and 8) were compared with the approved clinical results and are shown in Figs. 6 and 7. The Dp dose distribution results in the right lung are more consistent with the clinical results. Figures 8 and 9 present the 2D gamma analysis criteria of 2%/2 mm and 3%/3 mm with the Rp and Dp doses as references for the clinical dose. In Table 5, which summarizes the average gamma analysis passing rates calculated from all slices of the 2D dose with clinical dose distribution, there is no significant difference in the Dp model criteria of 2%/2 mm, and the passing rate of 0.03 is high at 3%/3 mm. In particular, the standard deviation of the Rp model is approximately two times higher than that of the Dp model.

**Table 5:** All of the slice 2-D gamma analysis average pass rate with 3%/3 mm and 2%/2 mm criteria for 10 test sets

Dosimetric index	2%/2 mm		3%/3 mm	
	Dp	Rp	Dp	Rp
Ave	0.71	0.70	0.85	0.82
SD	0.06	0.12	0.04	0.10

**Fig. 10:**
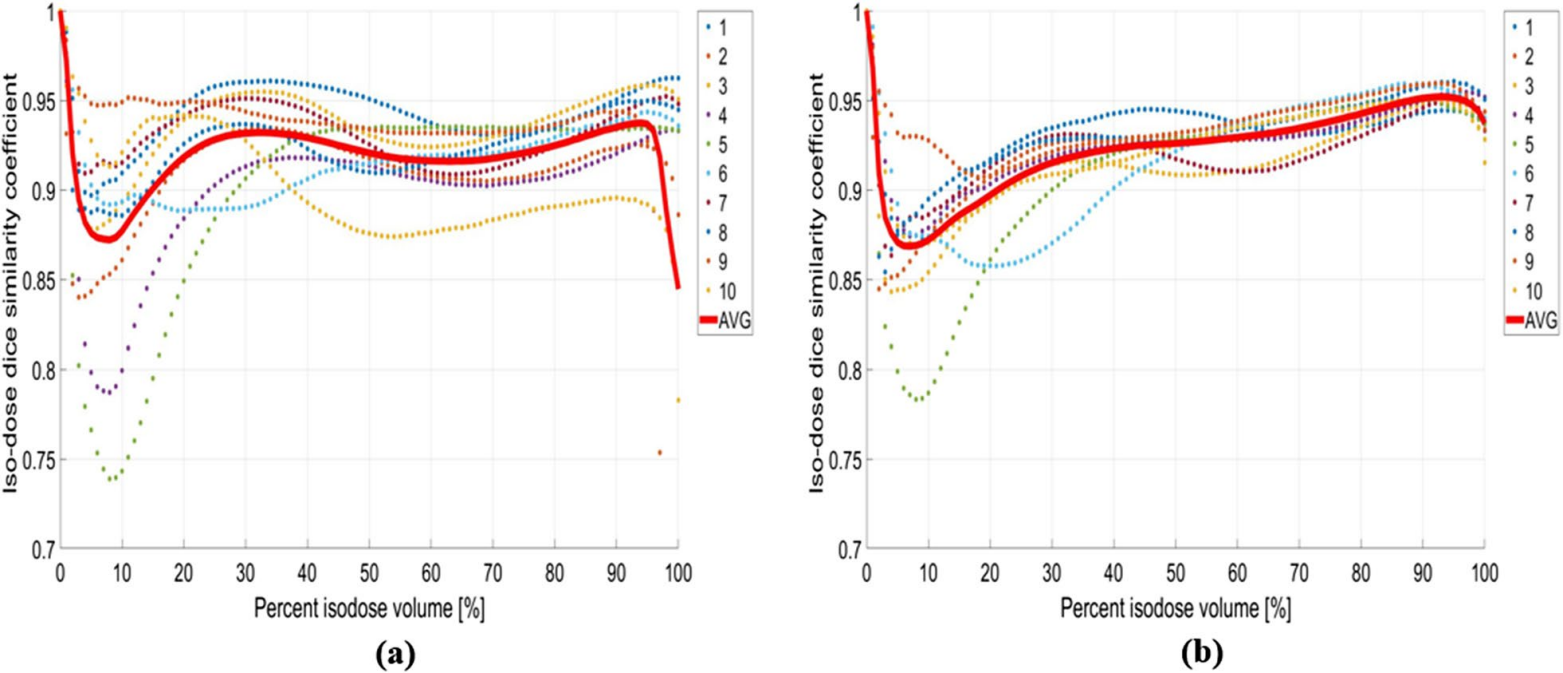
Iso-dose dice similarity coefficient (iDSC) between clinical and predicted isodose volumes (Dp and Rp) for ten test sets. **a** result of Rp iDSC, **d** result of Dp iDSC

Figure 10 displays iDSC for 10 test datasets from 0 to 100% of the isodose volume. The solid red line represents the average iDSC, which usually ranges from zero to one, with one indicating an ideal match. The Rp and Dp low isodose volumes (ranging from 3 to 20%) show a tendency for iDSC to be less than 0.9. The Dp model always has iDSC > 0.9 at a high isodose volume (range from 90 to 100%). However, the Rp model has cases in which the iDSC of the test set (Nos. 2, 9, and 10) is less than 0.9.

## Discussion

In this study, deep learning was utilized for VMAT dose distribution prediction using the anatomical features in the planning CT for left-sided breast cancer and the performance of this approach was compared with that of RapidPlan.

Our DpNet model consists of convolutional neural network layers to output the dose distribution according to the input target, OAR contours, and anatomical information (CT) (Fig. 1). Deep learning-based dose prediction studies have been reported on tumors centrally located in the body, such as rectal [24] and prostate [25, 26] cancer tumors. In the case of breast cancer, the target anatomical position is close to the body outside the area and the left lung, and the dose conformity is lower than that in the prostate case [41, 42]. The approach using deep learning to predict the clinically accepted dose distribution in the case of inhomogeneity around the target is different from those utilized in previous studies [24–26]. In Table 6, Yoganathan and Zhang [43] predicted the dose distribution for left breast cancer and reported that the prediction D_mean_ error (over entire CT volume) was 0.9 ± 1.2 Gy with an atlas-based method. Bai et al. [44] obtained 0.48 ± 2.27 Gy and 0.42 ± 1.82 Gy from dose prediction using the similarity selection method (SIM) based on the most similar atlas image and the weighted method (WEI_F) applying weighted dose distribution with database images.

**Table 6:** Summary of MADs presented in previous literature on left breast cancer

	Yogananthan et al. [40]	Bai et al. [41]		Our study	
Training data set	19	20		50	
Method	A	A (SIM)	A (WEI_F)	D (Dp)	A (Rp)
MAD (Gy) over entire CT volume	0.9 ± 1.1	0.48 ± 2.27	0.42 ± 1.82	0.32 ± 0.07	0.35 ± 0.08

A: atlas-based dose prediction, D: deep learning-based dose prediction

The mean absolute differences (MADs) [43, 44] in the test set over the entire CT image according to the Dp and Rp models show small differences from the clinical plan dose, and the SD is also small, indicating consistent dose prediction.

As the patient datasets and breast cancer treatment protocols differ by institution, the ability to perform direct comparison is limited; however, evidence has shown that dose prediction using deep learning methods is possible. Tables 2–4 demonstrate that the Dp model obtained results superior to those of the Rp model because it differed less from the clinical plan. In particular, in Fig. 10, the Dp model shows iDSC < 0.9 only in the region of low iso-dose volumes (from 2 to 20%), but for the Rp model in cases Nos. 2, 9, and 10, the results are less than 0.9 at high iso-dose volumes (from 95 to 100%) as well as low iso-dose volumes.

The calculated iDSC was less than 0.9 at high iso-dose volumes because the process of obtaining the optimal treatment plan using the Rp model optimization process and the manual method are different [45].

If the plan created using the Rp model does not satisfy the clinical goal in terms of the DVH and dose distribution, re-optimization must be performed, which is time consuming.

The time efficiency was determined based on the average times required by the Dp and Rp models for dose prediction, which were 9.82 ± 0.37 s and 676.08 ± 81.23 s, respectively (i.e., the times differ with statistical significance because the p-values were smaller than 0.05 when a ranked Wilcoxon test was performed).

The reason for why the time difference in the process in the dose prediction of the two models was large is that Rp spends a large amount of time on MLC (multi leap collimator) optimization and final dose calculation. On the other hand, in the case of Dp, dose prediction is possible in seconds due to the fact that only image information is required as an input, while model training takes a long time.

In this study, since the goals of the models of Dp and Rp are different, it is difficult to accurately compare the performance. Rp can create a treatment plan, and it is also possible for the planner to directly intervene to create treatment goal or better plan. However, in the case of Dp, only 3D dose prediction in our model it is possible, and for a better plan, the process of training and validation of the model is required again. However, other studies only compared the clinical results [22–30] The Rp model was used to compare the dose prediction accuracy of the Dp model for the same patient test case.

The reason for the deviation in the prediction result of the Rp model may be due to the difference between the quality of the input plan and the dosimetric strategy for Rp model training, as suggested by Fogliata et al. [46].

As shown in Figs. 8 and 9, the potential cause of the difference from the clinical result in the gamma analysis result in the right breast region is also a result of the imbalance in each plan objective.

This study had several limitations.

First, the current deep learning model provides only patient anatomical information (CT, contour) as inputs without dosimetric information. Second, only the learned one-type dose prediction is possible, and the deep neural network must be retrained for other IMRT or 3D-CRT treatment techniques and other sites.

Third, it is impossible to perform conversion into an executable treatment plan using the predicted dose distribution results.

Fourth, when selecting the plan data used to train the Rp plan, it is necessary to evaluate and confirm each plan by considering the difference between each primary object.

Nevertheless, dose predictability using deep learning was demonstrated in this study by quantitatively comparing the patient dose predictions obtained by automated radiation treatment planning [47] using deep learning with those determined existing commercial programs. The dose distributions predicted by deep learning will help reduce the iterative optimization process because planners can identify the areas in which to deliver increased or decreased doses in advance.

In a future study, to overcome the limitations of the currently developed Dp model, the dosimetric feature [24, 30] should be included in the input data to reflect the physical characteristics to increase the dose prediction accuracy. Based on the learned model, transfer learning [21, 48] will be applied to enable dose prediction for various treatment sites.

We will be able to evaluate the accuracy of the plan and the time it takes for the planning time after conducting a test on the new patient to see if the proposed model can provide effective guidance in order to satisfy the goal of the treatment plan.

We will develop a program using the Eclipse Scripting API to generate an optimal plan automatically based on the predicted dose.

## Conclusion

In this study, VMAT dose distribution predictions obtained by deep learning were compared with RapidPlan results for left-sided breast cancer patients using contour and CT images only. Our deep learning model produced superior dose predictions compared to RapidPlan and showed that dose prediction using deep learning is possible. In addition, radiation treatment planning based on the dose predicted using deep learning will improve the radiation treatment process by reducing the time required for planning, while maintaining plan quality.

## Data Availability

The data are not publicly available because of patient privacy concerns but are available from the corresponding author upon reasonable request.

## Abbreviations

AAA: Analytical anisotropic algorithm
CT: Computed tomography
CNN: Convolutional neural network
DpNet: Dose prediction deep neural network
DVH: Dose volume histogram
Dp: Deep learning dose prediction
ESAPI: Eclipse scripting API
iDSC: Isodose volume dice similarity coefficient
IMRT: Intensity modulation radiation therapy
KBP: Knowledge-based planning
MAE: Mean absolute error
MSE: Mean squared error
MAD: Mean absolute difference
OAR: Organs at risk
PTV: Planning target volume
ReLu: Rectified linear unit
RT: Radiotherapy
Rp: RapidPlan dose prediction
SD: Standard deviation
VMAT: Volumetric arc radiation therapy
